# Outcome of surgical management of bony metastases to the humerus and shoulder girdle: a retrospective analysis of 93 patients

**DOI:** 10.1186/1477-7800-3-5

**Published:** 2006-03-01

**Authors:** Duy M Thai, Yasuyuki Kitagawa, Peter FM Choong

**Affiliations:** 1 Department of Orthopaedics and University of Melbourne Department of Surgery, St. Vincent's Hospital Melbourne, Australia; 2 Division of Surgical Oncology, Peter MacCallum Cancer Institute, Melbourne, Australia

## Abstract

**Background:**

Metastatic disease to the shoulder girdle is a challenging problem because of the potential for pain, pathologic fracture and loss of function of that limb. Management of the bone disease centers around palliation, prevention of further complications and the preservation of residual function. A variety of surgical options exist for managing metastatic disease to the shoulder girdle and our experience with over 90 patients is reported. We focus on a preferred technique of combining rigid intramedullary nailing with cementation.

**Methods:**

Patients with metastatic disease to the shoulder girdle were accrued over a 9 year period from 1996 to 2004. 93 patients were identified with 96 operations being performed. The median age was 63 years (range 33 – 89) and 54% were female. The commonest primary tumor to metastasize was breast, and the proximal and midshaft humerus was involved in 84% of cases. The median survival time was 8 months and at last review 82% of patients had died of their disease

**Results:**

Operations performed were intramedullary nailing (n = 51), resection with or without prosthetic reconstruction (n = 34) or plate osteosynthesis (n = 9). The site of the metastasis was a guide to the most appropriate operation. Amputations (n = 2) were not done as the primary procedure.

Median post operative hospitalization ranged from 3 to 6 days depending on the type of operation performed. Our preferred technique for diaphyseal lesions (intramedullary nailing plus cementation) achieved excellent results in terms of pain relief, functional restoration and minimal complications. Functional restriction was most notable for proximal humeral prostheses (35% of patients).

**Conclusion:**

Surgical treatment of metastases to the shoulder girdle can be successful, allowing prompt relief of pain and return to prehospital level of care. Proximal and midshaft humeral metastases are easily amenable to resection and reconstruction or intramedullary nailing with cementation. Relief of pain and preservation of function occurs for the majority of patients.

## Background

Metastatic disease is the most common malignant bone tumor [1], with the humerus being the second most common long bone to be involved after the femur [1-3]. This can result in pain, loss of function and pathological fracture. The healing potential of pathologically fractured bone is low, hence the need for operative intervention in many of these patients. A functional upper limb is pivotal to a patients' independence, therefore, preserving limb function is a major goal of treatment.

A review of the literature over the past 20 years has shown a lack of published studies with adequate numbers of patients. Lewallen *et al *[4] reported their outcomes on 54 patients with humeral fractures in 1982. Our study, the largest to date, examines the different types of operative treatments for metastatic disease to the upper limb in 93 patients. Specifically, we have focused on a preferred technique of augmenting rigid intramedullary fixation with cementation.

## Patients and methods

Patients with metastatic disease to the humerus who underwent operative treatment were identified from the audit database of the Department of Orthopedics at St Vincent's hospital, Melbourne, a tertiary referral centre for the management of bone and soft tissue tumors. The setting is retrospective and the information was mainly from case records. Bony metastases to the humerus are rare and running a prospective study for many years would be difficult.

The patients were accrued over a 9 year period, from Jan 1996 to Dec 2004. Information was gathered from the medical records and this included patient demographics, site of metastasis, primary tumor, operative details (including length of operation), length of post operative stay and any complications. Outcome measures of pain and function were assessed subjectively by the patients and recorded by the examiner at the time of review. The data was obtained from the most recent clinical review appointment documented in the history. Because of the variability in accurately assessing degree of severity of the pain, we chose to record patients as either having no pain or pain which was causing them discomfort. Patients had restricted function if they were unable to use the affected limb in the usual manner.

### Patients

93 patients were identified from our database with 96 operations being performed. 2 patients required revision surgery and 1 had bilateral humeral metastases which were operated on. The median age was 63 (range: 33 – 89) years with 54% female.

### Presenting symptoms

Pathologic fracture was the commonest presentation (52 patients, 56%). Pain without fracture was the main presenting complaint in 41% of cases. Only 2 patients presented with a painless lump in the arm.

### Tumour type and location

**Figure 1:**
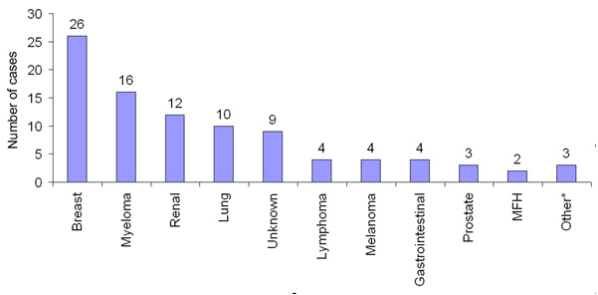
**Primary tumors. **Primary tumors which have metastasized to the shoulder girdle. The number above the columns represents number of cases.

Breast carcinoma was the commonest histotype with almost 25% of patients presenting with this diagnosis. Myeloma, renal and lung carcinoma were the next most common diagnoses (Figure 1). Almost 90% of tumors were located in the diaphyseal (middle third) or proximal (proximal third) humerus (47% and 42% respectively). Distal humeral (distal third) metastases were uncommon, with 11% involvement. Scapular and clavicle involvement was rare (4% and 2% respectively)

### Surgery

The most common procedure was the insertion of a rigid intramedullary rod and the second most common procedure was resection of the metastatic lesion and prosthetic reconstruction (Table 1). Plate osteosynthesis was predominantly used for diaphyseal and distal humeral lesions. The location of the tumor in the humerus was a significant determinant of the type of operative procedure performed.

**Table 1:** Relationship of operations performed to the site of metastses

	Proximal humerus	Midshaft humerus	Distal humerus	Clavicle	Scapula	Total
Intramedullary rod	14	37	0	0	0	51
Plate and screws	0	2	6	1	0	9
Resection & prosthesis	22	2	4	0	2	30
Resection alone	2	0	0	1	1	4
Amputation	1	1	0	0	0	2
						96

**Figure 2:**
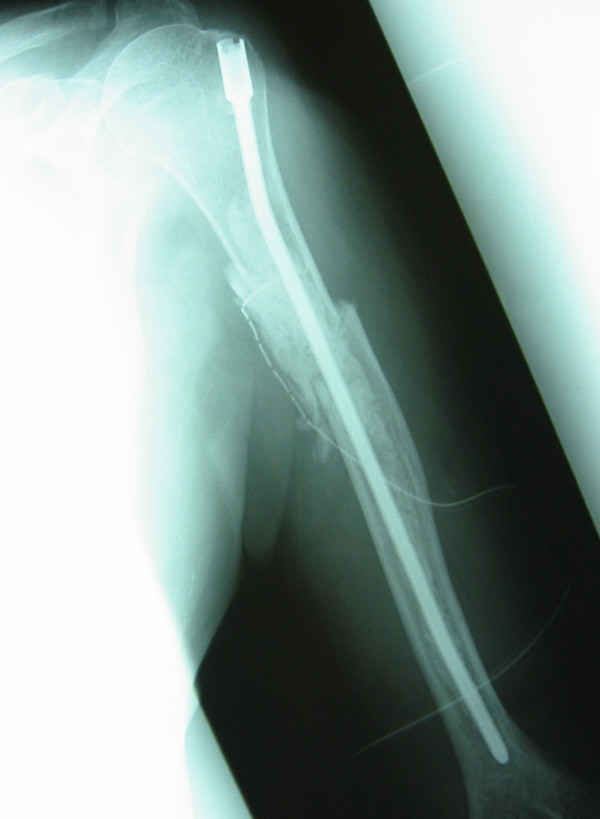
**Radiograph of Alta humeral nail. **Post operative radiograph of the Alta humeral nail inserted for treatment of a pathological fracture of the humeral diaphysis. Cement can be seen in situ around the nail which is injected via the proximal entry hole prior to insertion of the nail.

Intramedullary nailing was performed in 51 cases mainly for diaphyseal lesions and in some proximal lesions where there was sufficient normal proximal bone for the rod to span. The Alta intramedullary rod (Stryker Howmedica Osteonics, Mahwah, NJ) with methylmethacrylate bone cement (Edurance, DePuy, Warsaw, IN) was used in 44 cases. The Alta rod was chosen because of its fluted design which allows backflow of cement on insertion (Figure 2). 4 cases used the Fixion inflatable rod (Disc-O-Tech Medical Technologies, Herzliya, Israel). The remaining 3 cases each utilized another type of rigid intramedullary rod with interlocking screws. The choice of rod used was determined by the operating surgeon. 8 cases were done via an open technique, with curettage of the tumor cavity and reduction of the fracture. The open technique was used if the fracture was unable to be reduced closed (due to comminution or marked displacement) or if the fracture or bone defect is adjacent to the path of the radial nerve as it winds posterior around the humeral shaft. By formally exposing the fracture, the nerve is able to be protected from potential cement injury and good alignment of the fracture can be maintained when inserting the rod and curing of the cement.

Prior to insertion of the intramedullary rod, the humeral canal was reamed and irrigated. A suction catheter was placed in the canal and low viscosity cement with Gentamycin antibiotic (Endurance, DePuy, Warsaw, IN) was injected into the proximal entry hole. The intramedullary rod was inserted while the cement was still wet in order to reduce the risk of pulmonary embolic phenomenon [5,6].

Recognizing that the tumors were metastatic carcinomas, and in all cases, treatment was for palliation, the aim of surgical margins when inserting a prostheses was to preserve as much bone as was reasonably possible and also to preserve and reconstruct the rotator cuff. A variety of prostheses were used with cementation of the stem to the shaft. Proximal prostheses were used if there was involvement of the humeral head or insufficient healthy bone to allow proximal purchase of an intramedullary rod. There were 20 Isoelastic proximal humeral replacements (Mathys, Bettlach, Switzerland) and 2 Neer II (Smith and Nephew, Mansfield, MA) prostheses. Not all of the proximal humeral prostheses have the facility to repair the residual rotator cuff to the prosthesis. If suture holes are available to reconstruct the capsule or rotator cuff to the prosthesis, then this should be done to enable greater stability at the shoulder. If no suture holes are available, then nylon mesh fixed around the proximal part of the prosthesis can be used as a reliable anchoring site onto which capsular or rotator cuff fixation may be performed. Stabilizing the shoulder allows more useful function of the hand and elbow, and decreases the "dragging sensation" that some patients experience with inferiorly subluxing prostheses.

2 intercalary prostheses were used for diaphyseal lesions. Distal lesions treated with prosthetic reconstructions used a total elbow replacement system (Zimmer, Warsaw, IN). Scapular lesions were treated with scapulectomy in conjunction with proximal humeral resection and reconstructed using scapular prostheses with a proximal humeral prosthesis.

Plate and screw fixation was performed after curettage, dental burring, treatment with phenol and alcohol and finally, cementation of the bone defect. This was performed mainly for distal lesions. Plate osteosynthesis for diaphyseal lesions was only performed in 2 cases.

Amputations were not done as primary procedures. The 2 cases involved were due to complications of previous intramedullary nailing. One patient had extensive local tumor compression of the axillary neurovascular bundle 6 months following the primary procedure, resulting in gangrene of the arm. The other patient had a previous intramedullary rod which was causing persistent pain. The primary tumor involved was breast and renal carcinoma respectively.

## Results

**Figure 3:**
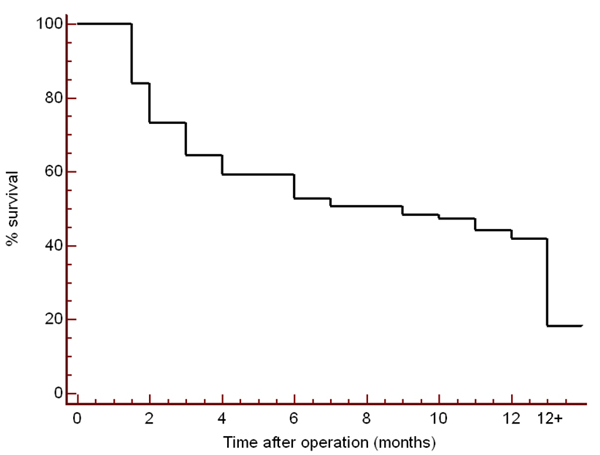
**Survival curve. **Kaplan-Meier survival curve for patients with metastases to the shoulder girdle in the first 12 months after operation.

Of the 93 patients, 76 (82 %) had died of disease. The remaining 17 patients were alive at the time of the study or no mortality data was available. Mortality data was obtained from the patients' medical records or from the State registry of births, deaths and marriages. The mean length of follow up for the patients who were still alive was 31.7 months (The longest period of follow up being 58 months). The mean survival time of the patients who died was 13.2 months with a maximum of 51 months. 50% of patients were still alive 8 months after surgery (Figure 3).

### Operative time and length of stay

The operative time was recorded from the time of skin preparation to application of the dressing. Prosthetic reconstructions took the longest operative time for completion (median 150 minutes) while insertion of intramedullary fixation was the quickest (median 75 minutes). A similar pattern was noted for length of acute hospital stay. The longest median time for hospital stay following resection and prosthetic reconstruction was 6 days compared to a median of 3 days for intramedullary nailing.

### Pain control and function

Pain control was very good after intramedullary rod insertion and prosthetic reconstruction with only 8% and 13% respectively of patients having any persistent post-operative pain (Table 2). Restriction of any function was the highest after prosthetic replacement (35% for proximal prostheses, see Table 2).

**Table 2:** Outcome versus procedure

	*n*	Persistent pain	Restricted function	Revision	Death prior to review	No data
Intramedullary rod	51	4 (8%)	3 (6%)	1 (2%)	16 (31%)	8 (15%)
Plate osteosynthesis	8	1 (13 %)	1 (13 %)	0	3 (38 %)	0
Proximal prosthesis	22	3 (13%)	8 (36 %)	0	2 (9%)	5 (22%)
Midshaft prosthesis	2	0	0	0	0	0
Distal prosthesis	4	1 (25%)	1 (25%)	1 (25%)	0	0
Resection alone	2	No data	1 (25%)	0	1 (25%)	2 (50%)
Amputation	2	1 (50%)	NA	NA	0	0

### Complications

Nerve palsy was the commonest complication (6 %) and the radial nerve was the one affected (Table 3). Another important and unexpected complication was pulmonary embolic phenomenon which was observed in two cases. Re-operations were performed in 2 patients (Table 2), one in a patient who had intramedullary nailing, the other in a patient who had a total elbow replacement for a distal humeral lesion. The first patient had local vascular invasion of the tumor resulting in gangrene of the arm necessitating amputation. The second patient had loosening of their prosthesis 2 years after the initial surgery and was changed to a long stem Conrad-Morrey elbow replacement (Zimmer, Warsaw, IN).

**Table 3:** Complications

Operation	Complication	*n*
Operation	Complication	*n*
	Nerve palsy	3
	Fat embolus	1
	Wound infection	1

Plate fixation	*n*	4
	Nerve palsy	2
	Fat embolus	1
	Plate loosening	1

Resection alone	*n*	1
	Wound infection	1

Proximal humeral resection	*n*	5
	Intraoperative bleeding	1
	Myocardial infarct	1
	Stitch abscess	1
	High riding prosthesis	1
	Proximal migration of prosthesis	1

Distal humeral resection	*n*	1
	Nerve palsy	1

## Discussion

Our results show that half of patients with metastatic disease to the upper limb remain alive at 8 months, with a mean of 13.2 months. These patients thus deserve adequate treatment of their disease despite being generally palliative. Adequate treatment should involve surgical intervention as conservative management (splints or casts) of these fractures has shown poor results. Fleming *et al *[7] demonstrated non union in 50% of cases and inadequate pain relief in 88% with conservative measures.

Pain, whether it be a result of a fracture or not, was a significant presenting symptoms in all but 2 of our patients. The surgical options addresses the primary aim of pain control, but also needs to be durable enough to allow early mobilisation in pathologically weakened bone to preserve limb function.

The majority of our patients had treatment for pathologic fracture (56%). Prophylactic treatment has been advocated by many [2,7,8] because it makes the operation technically easier, reduces the risk of complication and may also reduce the risk of developing systemic metastases [8]. We performed prophylactic surgery on 37 of 93 patients (39%) referred with persistent pain following radiation therapy. Measures to predict the risk of pathological fracture are available (Mirrels' scoring system [9]) but were not formally evaluated in this study.

We used predominantly rigid intramedullary nailing with cementation in the canal and this has been successful in our hands. It is chosen when there is sufficient normal proximal and distal bone for the rod to span. If this is the case, then it is highly likely that the rotator cuff will be left intact apart from a lateral entry point for the nail which is easily repaired. These patients may be reassured that their shoulder function can be mostly preserved. Only 8% had any persistent pain and 6% restricted function of the affected limb. We have been unable to find any comparable reports of this technique in the literature. There are numerous reports of flexible intramedullary nailing using Hackethal [10] or Rush rods [4] to good effect, however we believe that rigid intramedullary nailing provides a more durable and stable construct to allow early mobilization. An alternative to cementation is the use of rigid intramedullary nailing with interlocking screws. Dijkstra *et. al*. [2] reported 88% of patients achieving good pain relief and 94% with good function following this technique. However, there were 3 failures of fixation due to angulation, rotation and refracture. There were no failures of fixation using our technique. We used cement in conjunction with rod fixation for a number of reasons. These included strengthening the residual bone, enhancing fixation of the rod along its whole length not just at its ends, thus providing rotational stability [8,11,12], and also for the thermonecrotic effect of cement on tumor cells as the cement cures [11]. The need for cross bolting is obviated, minimizing the creation of new defects and thus weaknesses in the remaining bone.

From our series, 3 out of 51 cases (5.8%) of intramedullary fixation developed transient radial nerve palsy. Only 1 was from using the Alta nail with cement. The other 2 were from the Fixion inflatable nail. There were only 4 Fixion nails inserted in our series and the reason for the higher incidence of nerve palsies is unclear. The numbers are too small to draw any meaningful conclusions at this stage. There has been one report of the Fixion inflatable nail being used in 23 cases for pathologic fractures of the humerus in a series by Franck *et. al*. [13] with no complications. We believe that the fixation achieved by inflating the nail is not rigid enough to take into account the progressive nature of the metastatic deposit. This can potentially lead to loosening of the nail as the deposit enlarges.

Our incidence of radial nerve palsies (5.8%) is slightly higher than that reported from other series in which intramedullary nailing was used to treat non pathologic humeral fractures. Rommens *et. al*. [14] had a rate of 4.7% for retrograde nailing and Demirel *et.al*. [15] a rate of 3.5% for antegrade nailing. The higher rate in our study reflects the difficulties associated with tumour surgery, in particular scarring around the area as a result of radiotherapy and the soft tissue component of the tumour potentially involving the nerve.

Plate fixation should generally be avoided for diaphyseal lesions as the screws are put into structurally inadequate bone [16]. We performed only 2 in severely comminuted diaphyseal fractures. Dijkstra *et al *[2] showed no difference in terms of pain relief, function and complications between intramedullary fixation with interlocking screws versus plating and hence advocated either technique for use in midshaft fractures. However, they suggest that post operative radiation not be used for plate osteosynthesis due to osteoporosis and impaired bone healing, which is important for adequate screw fixation. The majority of our patients who were well enough underwent post operative radiotherapy to the affected area, which we believe is an important adjunctive treatment to the surgery.

The aim of treatment of proximal humeral lesions is to regain shoulder stability and pain relief. Functional results are usually poor due to extensive destruction of the rotator cuff soft tissues [16]. In our results, the outcome of proximal humeral resections and reconstructions was fair, with 35% reporting restricted function. Persistent pain was reported in only 13% of cases. In 2 cases the prosthesis was high riding, resulting in restricted mobility. Patients need to be made aware of the aims of treatment (pain relief and stability) and the likelihood of persistent shoulder stiffness after the operation prior to obtaining consent for this type of procedure.

Distal humeral lesions were managed predominantly with plate osteosynthesis but if there were more complex distal humeral fractures, then these required more extensive resection and reconstruction with total elbow replacements. Significant pain remained in half of our patients after elbow resection and reconstruction, but the few numbers make a firm conclusion difficult. There was only one nerve palsy in this group of patients (25%). Ross *et al *[17] reported a 31% incidence of nerve palsies in their series of custom built endoprothesis incorporating a hinged elbow to replace large distal humeral lesions.

## Conclusion

Metastatic disease to the upper limb should be treated surgically. The majority of patients will survive for a significant time after surgery and hence a stable and painfree limb should be the goal. Our preferred technique for diaphyseal lesions is rigid intramedullary nailing with methylmethacrylate cement inserted into the canal. This provides a durable and stable fixation allowing excellent pain relief, early mobilization and the potential for further adjuvant radiotherapy. Proximal humeral lesions are generally treated with resection and prosthesis, with good pain control but poor functional outcome. Patients need to be aware that a stiff shoulder may result from surgery in this area and the main goal of treatment is pain relief.

## Competing interests

The author(s) declare that they have no competing interests.

## Authors' contributions

DMT acquired the data with the help of YK, analyzed the data and drafted the manuscript. YK revised the manuscript. PFMC conceived of the study and critically revised the manuscript. All authors read and approved the final manuscript.
